# Radiolucent gallstone ileus causing proximal small bowel obstruction: A case report

**DOI:** 10.1016/j.radcr.2025.02.002

**Published:** 2025-03-15

**Authors:** Abdulmalek Alzahrani, Mohammad Alsayed, Jalal A. Zahhar, Saga Ali, Samira Alturkistany

**Affiliations:** a King Faisal Specialist Hospital and Research Center, Jeddah, Saudi Arabia; b King Saud bin Abdulaziz University for Health Sciences, Jeddah, Saudi Arabia

**Keywords:** Gallstone, Gallbladder, Ultrasound, Ileus, Radiolucent

## Abstract

Gallstone ileus occurs when gallbladder stones erode and become lodged in the small bowel, thereby causing obstruction. These stones usually impact the terminal ileum because of its narrow lumen; however, they can also be found in less common locations, as observed in our case. The presence of pneumobilia can indicate gallstone ileus even without visible radiopaque stones because not all gallstones are detectable on computed tomography (CT). A 74-year-old woman presented to the emergency department with severe right upper quadrant pain that started 1 week previously and became more aggressive associated with fever, leukocytosis, and vomiting. The pain was colicky, intermittent, and aggravated by movement but did not radiate. Further imaging was requested to investigate the cause of the pain, which revealed evidence of proximal small bowel obstruction due to radiolucent gallstone ileus. Radiolucent gallstone ileus is a rare but serious condition requiring prompt surgical intervention. The presence of pneumobilia can indicate gallstone ileus even when the stones are not visible on CT. Large stones can cause blockages in the proximal small bowel. The ability of radiological imaging to detect gallstones varies according to their composition.

## Introduction

Gallstone ileus is characterized by eroded gallbladder stones that are impacted in the small bowel, resulting in subsequent small bowel obstruction [1]. The eroded stones typically impact the terminal ileum because of its relatively narrow lumen; however, these stones can be seen in atypical sites, as in our case [1]. The presence of pneumobilia raises the suspicion of gallstone ileus even in the absence of radiopaque stones because not all gallstone types can be visualized on computed tomography (CT) [2].

## Case presentation

A 74-year-old woman presented to the emergency department with severe right upper quadrant pain that had begun 1 week earlier and progressively worsened. The pain was accompanied by fever, leukocytosis, and vomiting. She described it as colicky, intermittent, nonradiating, and aggravated by movement. On physical examination, the patient exhibited yellowish discoloration of the sclera (jaundice) and mild abdominal distention.

**Fig. 1:**
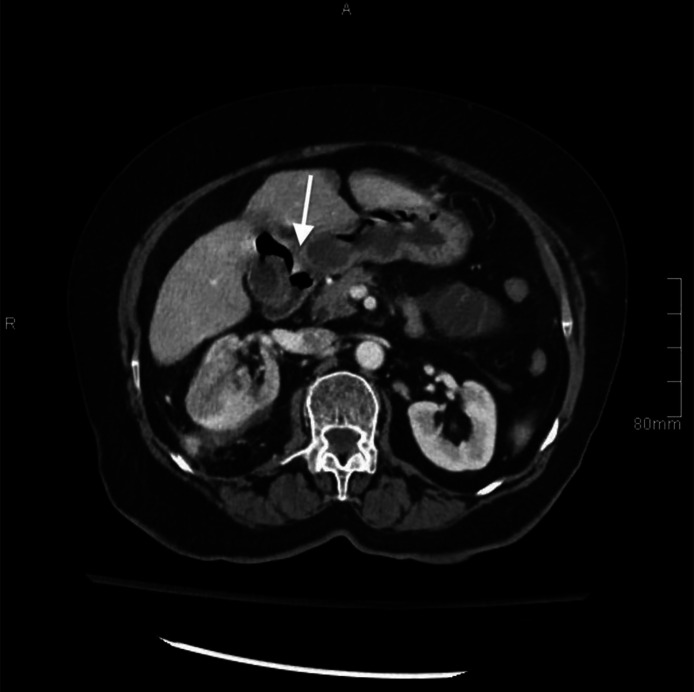
Axial contrast-enhanced CT image. The image shows a partially distended gallbladder with internal air foci representing fistulization of the first part of the duodenum (white arrow).

**Fig. 2:**
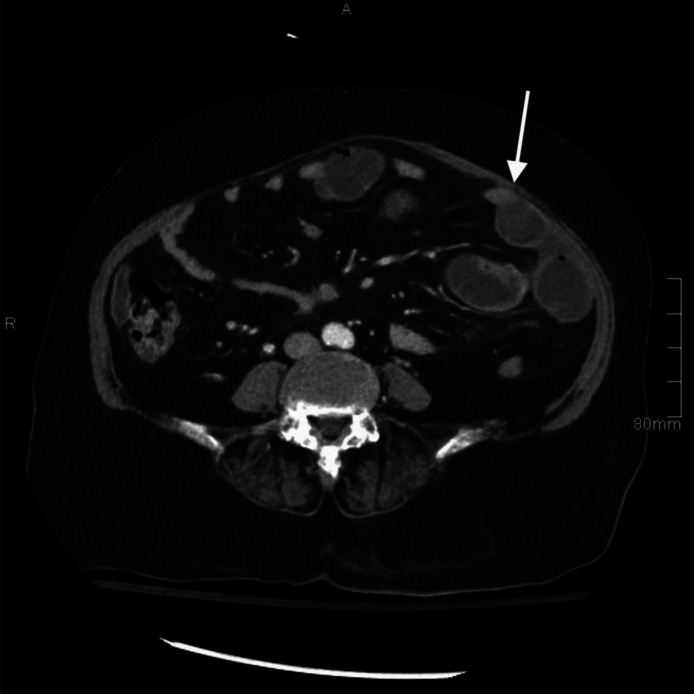
Axial contrast-enhanced CT image. The image shows a dilated mid-jejunal loop with a maximum diameter of 3.2 cm. A transition zone was observed at the distal jejunal loops (white arrow). Minimal pericholecystic-free fluid was noted. The overall findings raised the suspension of a radiolucent gallstone, eroded through a cholecysto-enteric fistula due to underlying chronic cholecystitis, with subsequent jejunal loop obstruction.

Due to the nonspecific clinical presentation, a contrast-enhanced CT scan was performed for further evaluation. The imaging revealed a large, dilated mid-jejunal loop with a maximum diameter of 3.2 cm (Fig. 1, Fig. 2) and a transition zone in the left anterior abdomen at the distal jejunal loops. Additionally, a partially distended gallbladder with internal air foci and a suspected fistulous connection to the first part of the duodenum was identified, along with pneumobilia. Notably, no hyperdense or calcified stones were observed.

**Fig. 3:**
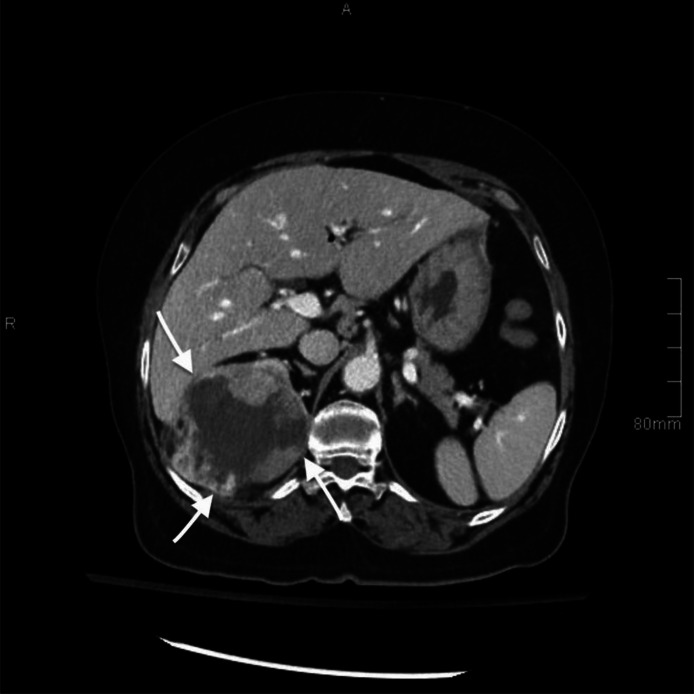
Axial contrast-enhanced CT image. The image shows a large mixed exophytic complex renal lesion in the right upper renal pole with an enhancing nodular component. The renal mass abuts the right hepatic lobe, diaphragmatic crus, and intercostal muscles (white arrows).

The CT scan also revealed an incidental finding of a mixed exophytic complex lesion in the right upper renal pole with an enhancing nodular component (Fig. 3). This lesion was located near the right hepatic lobe, the right diaphragmatic crus, and the intercostal muscles between the 11th and 12th right ribs. Furthermore, a filling defect was noted within the right renal vein.

**Fig. 4:**
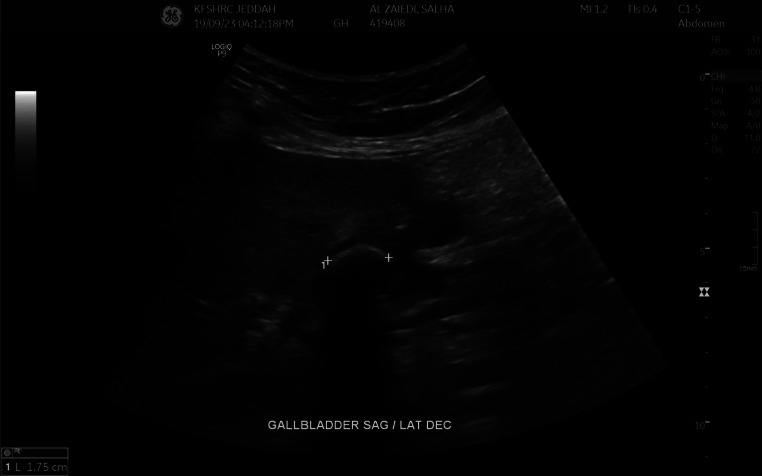
Grayscale ultrasound image. The image shows a large hyperechoic stone measuring 1.75 cm, showing posterior acoustic shadowing (white arrow); the stone was not visible on CT scan.

**Fig. 5:**
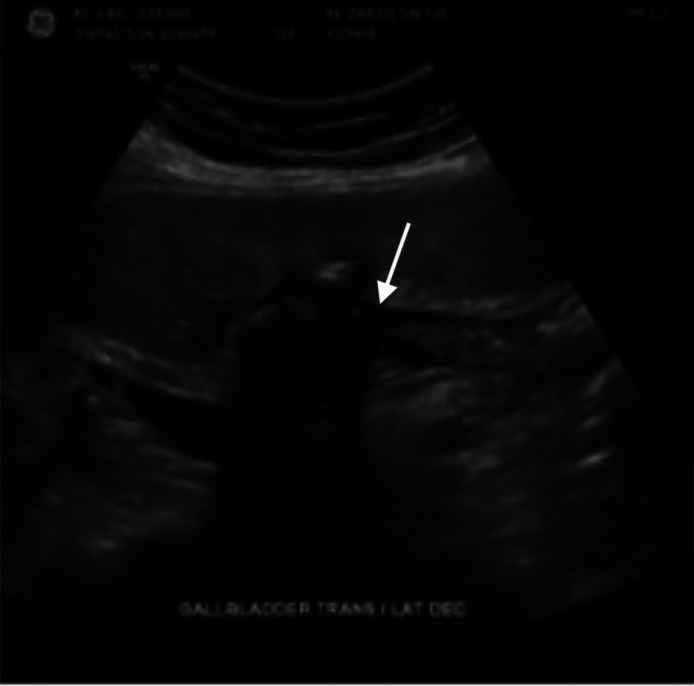
Grayscale ultrasound image. The image shows the choicysto-enteric fistula with the duodenum (white arrow).

Hepatobiliary ultrasonography was recommended to confirm the suspected cholecystoduodenal fistula. The ultrasound demonstrated a gallbladder wall defect and a 1.7 cm gallstone with posterior acoustic shadowing and a twinkle artifact (Fig. 4, Fig. 5).

**Fig. 6:**
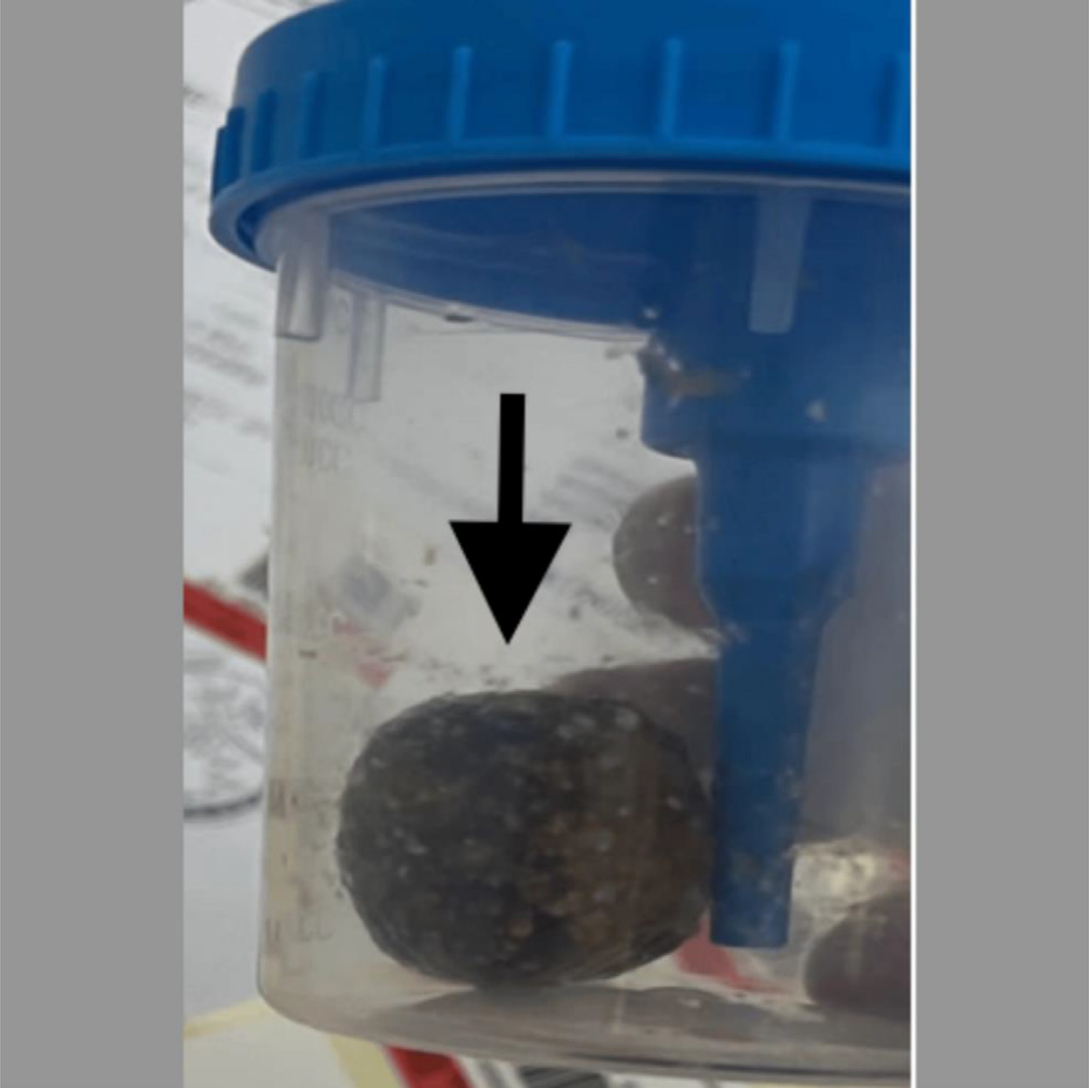
Postoperative specimen. The image shows a large gallstone retrieved from the mid-jejunal loop (black arrow).

The patient underwent an exploratory laparotomy, which confirmed the CT findings. A gallstone ileus was managed through stone retrieval via distal jejunal enterotomy, followed by primary closure. The patient had an uneventful hospital course and advanced to a regular diet by the third postoperative day, with no nausea, vomiting, or other complications. She passed bowel motions and voided freely. The patient was discharged (Fig. 6).

Several weeks later, the patient was readmitted for an elective radical nephrectomy. During the procedure, hepatobiliary surgeons were consulted. Intraoperative findings included a large gallstone occupying the gallbladder, a cholecystoduodenal fistula involving the first part of the duodenum, and adhesions of small bowel loops from the prior surgery. The patient underwent open right radical nephrectomy with inferior vena cava (IVC) thrombectomy, cholecystectomy, excision of the cholecystoduodenal fistula, and pyloroplasty.

Pathology findings were as follows:

1. IVC Thrombus Biopsy: Positive for carcinoma.
2. Right Kidney Nephrectomy: Clear cell renal cell carcinoma, Grade 4, with pathologic stage classification (pTNM, AJCC 8th Edition): pT3b.
3. Gallbladder (Cholecystectomy): Chronic cholecystitis, negative for malignancy.
4. Duodenal Fistula: Fragments of fibromuscular tissue with focal inflammation, old hemorrhage, and fibrosis, negative for malignancy.

## Discussion

Adhesions are the main causes of intestinal obstruction; however, some uncommon entities, such as gallstone ileus, may be interesting to investigate. Gallstone ileus is characterized by stone erosion from the gallbladder. These stones are impacted in the small bowel, resulting in subsequent small bowel obstruction [1]. The eroded stones are typically impacted at the terminal ileum because of its relatively narrow lumen; however, they can be seen in atypical sites, as in our case [2]. The presence of pneumobilia can increase the suspension of gallstone ileus, even when the stones are not visible on CT scans. Although mechanical intestinal obstruction due to intraluminal gallstones is considered relatively rare, it can be life-threatening, particularly in elderly patients with comorbidities [1,2]. The mortality rate is 12%–27%, and the morbidity rate is 50% [2]. Gallstone ileus has a female predilection, with ratios ranging between 3:1 and 16:1 [3]. The underlying pathology of gallstone ileus is a sequala of repeated cholecystitis, which leads to erosion and fistula formation in adjacent small bowel loops [3,4]. Surgical intervention is the mainstay of treatment [4,5].

## Conclusions

Radiolucent gallstone ileus is a rare but severe condition that should be diagnosed and treated promptly through surgery. The presence of pneumobilia can suggest gallstone ileus, even when the stones are not visible on CT. Large enough stones can cause blockage in the proximal small bowel loop. The effectiveness of radiological imaging in the detection of gallstones depends on their composition. Considering gallstone ileus as a potential cause of intestinal obstruction is important, particularly in elderly female patients.

## Data statement

The data presented in this study are available on request from the corresponding author due to privacy.

## Declaration of generative AI in scientific writing

None.

## IRB approval

The study was approved by the IRB committee

## Patient consent

Informed consent was obtained.

## CRediT authorship contribution statement

**Abdulmalek Alzahrani:** Investigation. **Mohammad Alsayed:** Writing – review & editing, Writing – original draft. **Jalal A. Zahhar:** Data curation. **Saga Ali:** Resources. **Samira Alturkistany:** Supervision.

## Appendices

Teaching Point

Radiolucent stones pose a diagnostic challenge because they are not visible on standard CT; therefore, identifying secondary features, such as pneumobilia, dilated bowel loops, and fistula, is crucial. Awareness of the limitations of each imaging modality is essential for accurate diagnosis. Note that the effectiveness of radiological imaging in detecting gallstones varies based on their composition, and the entire bowel should be thoroughly evaluated on imaging studies because gallstones can cause obstructions at sites other than the terminal ileum.
